# Supplementary material for: Antarctic Ice Sheet and emission scenario controls on 21st-century extreme sea-level changes
Source: Nat Commun. 2020 Jan 20;11:390. doi: 10.1038/s41467-019-14049-6 (PMC6971016; doi:10.1038/s41467-019-14049-6)
Supplement: Supplementary file 1 — Supplementary Information [file 41467_2019_14049_MOESM1_ESM.pdf]

Supplementary information for ’Antarctic Ice Sheet and emission scenario controls on 21st-century extreme sea-level changes’

Frederikse et al.

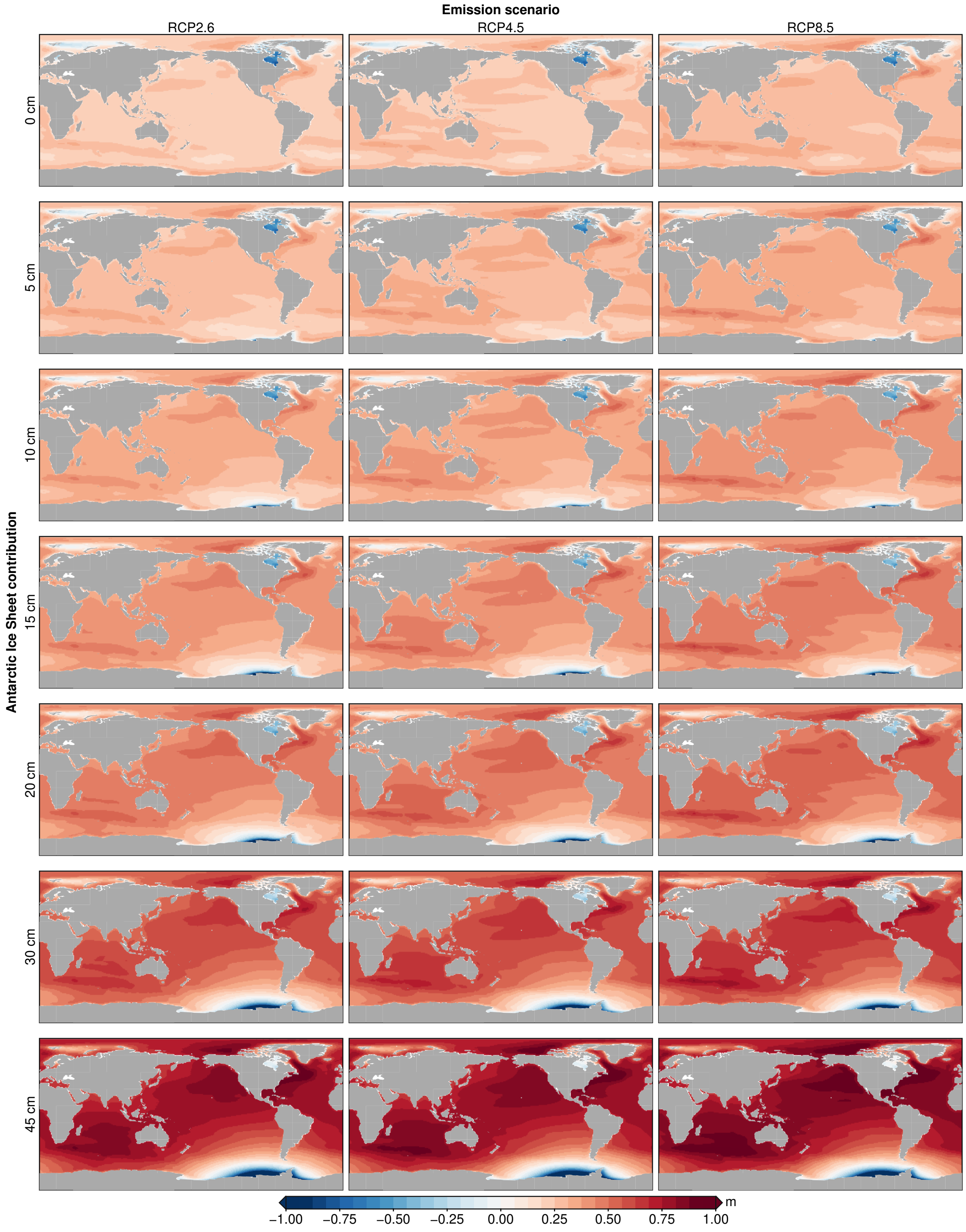

Supplementary Figure 1. Mean sea-level change scenarios for 2046-2065. All scenarios are relative to the 1986-2005 baseline.

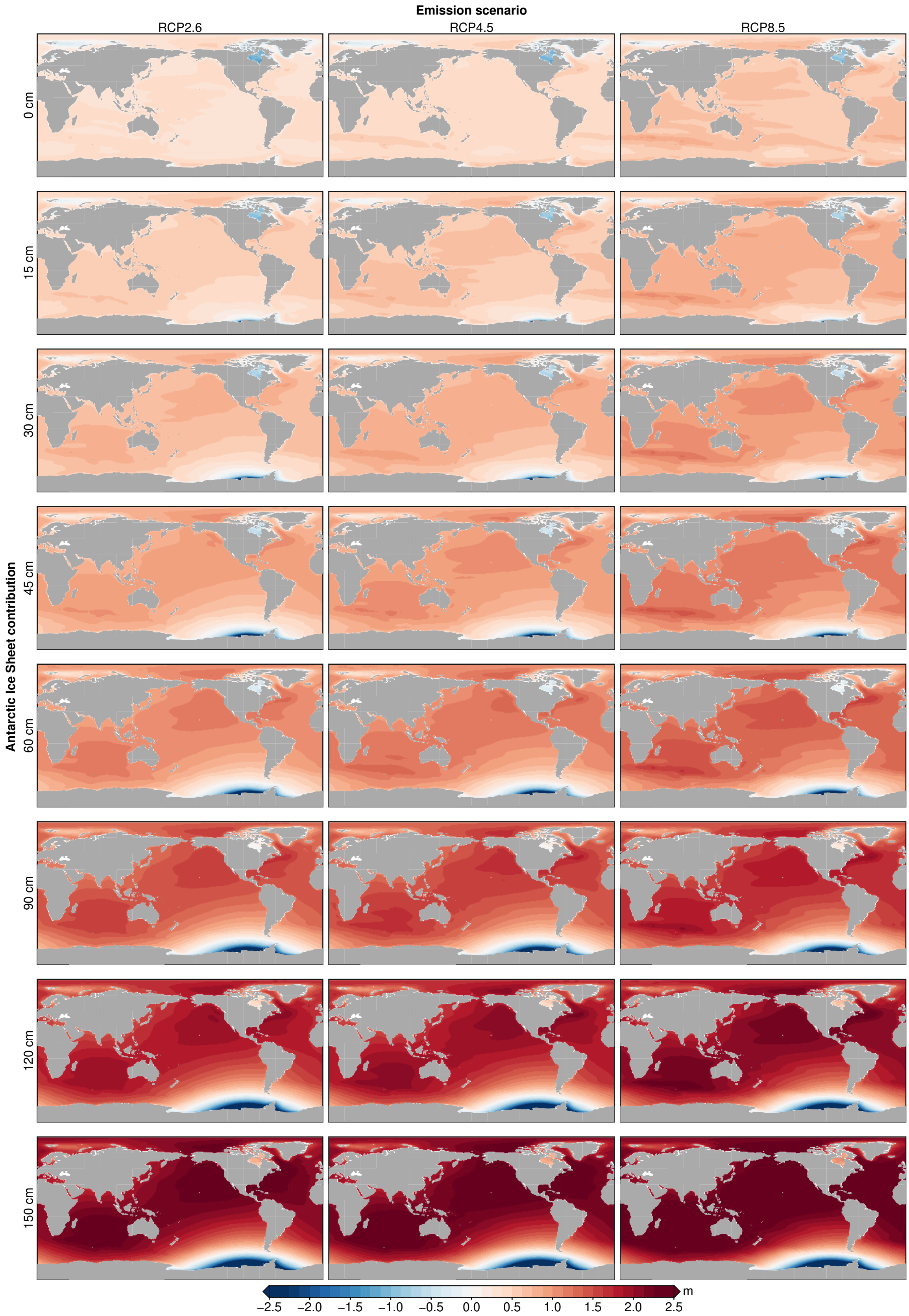

Supplementary Figure 2. Mean sea-level change scenarios for 2081-2100. All scenarios are relative to the 1986-2005 baseline.

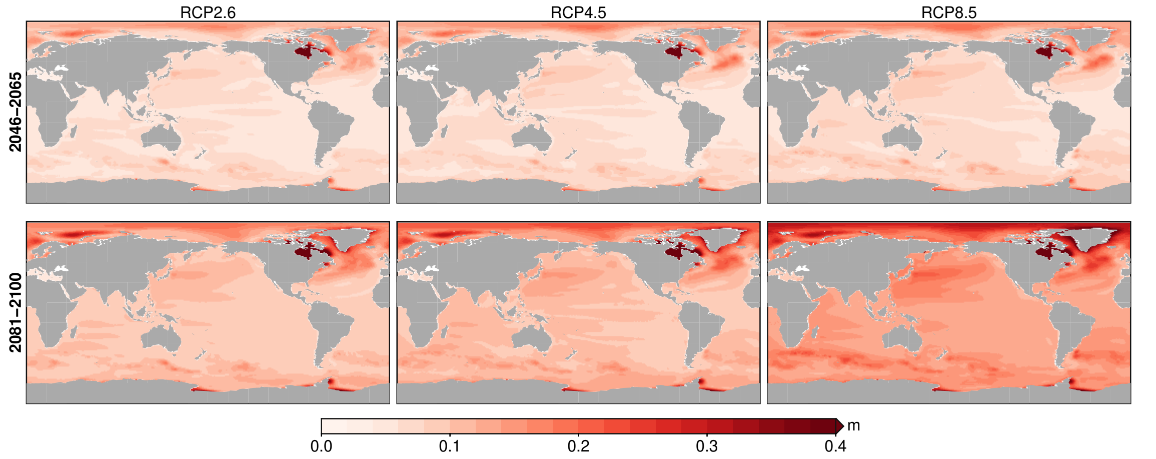

Supplementary Figure 3. Local 1σ uncertainties of the sea-level projections. Since we do not associate an uncertainty to each AIS scenario, these estimates do not contain an AIS component

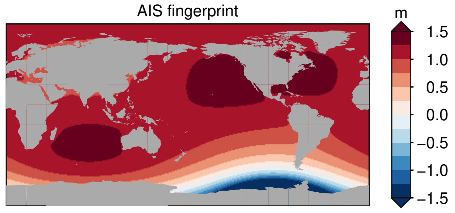

Supplementary Figure 4. Sea-level fingerprint for mass loss at the Antarctic Ice Sheet. Local sea-level change associated with a mass loss of 1 meter equivalent global-mean sea level. The mass loss is assumed to occur at Thwaites Glacier.

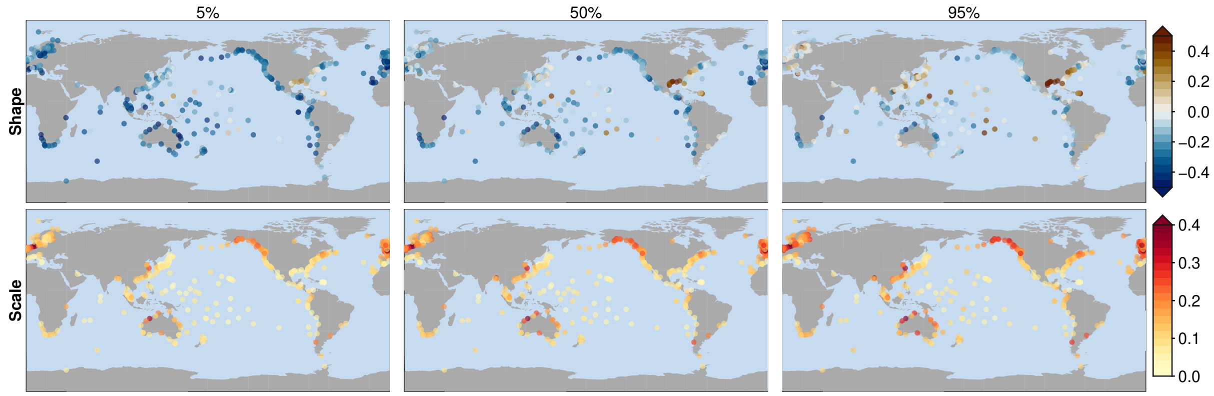

Supplementary Figure 5. The Generalized Pareto Distribution parameters. Shown are the median and 90-percent confidence intervals for the GPD shape and scale parameter for all sites.

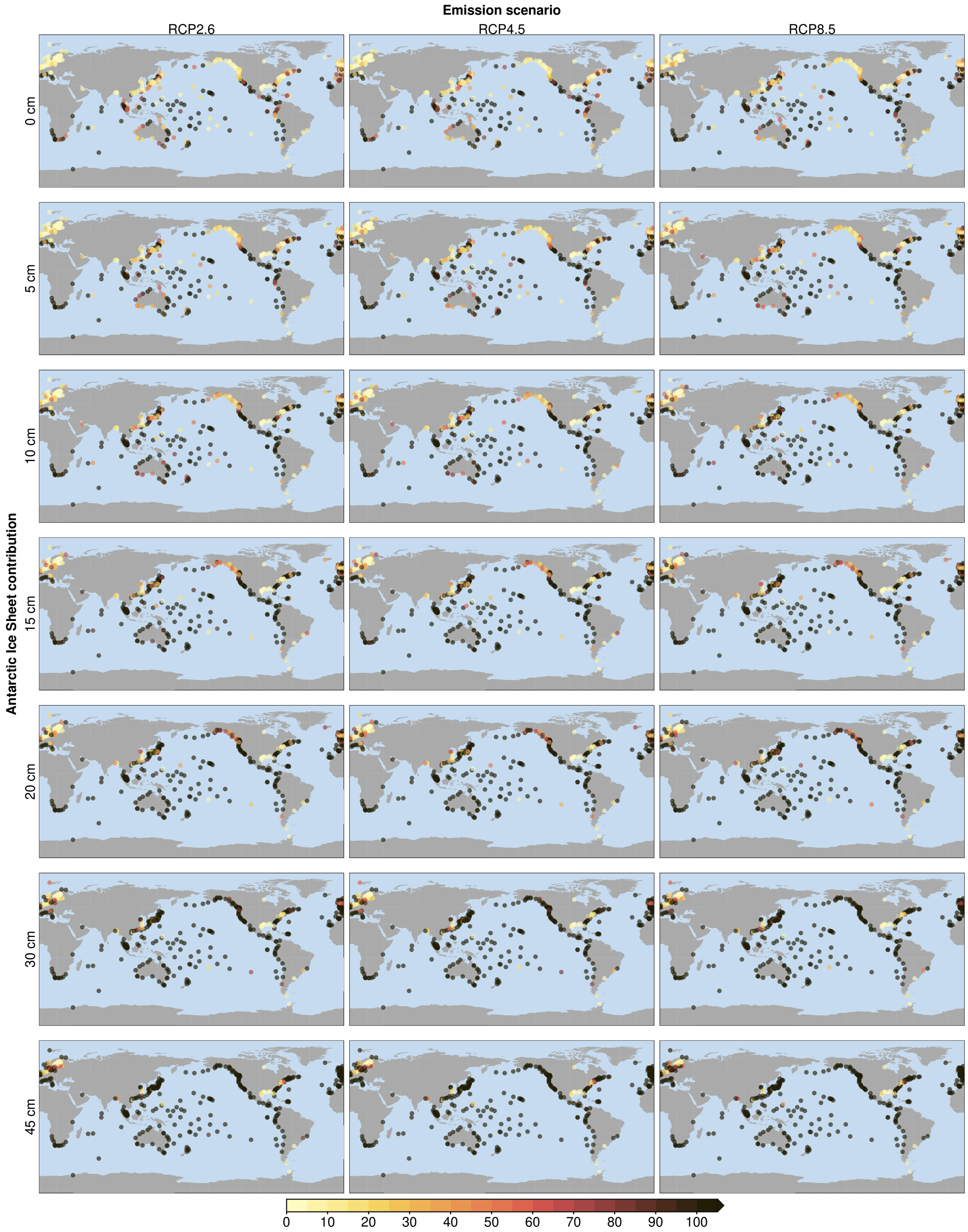

Supplementary Figure 6. Frequency amplification factors for the present-day 100-year ESL event in 2046-2065 for the RCP scenarios and AIS contributions.

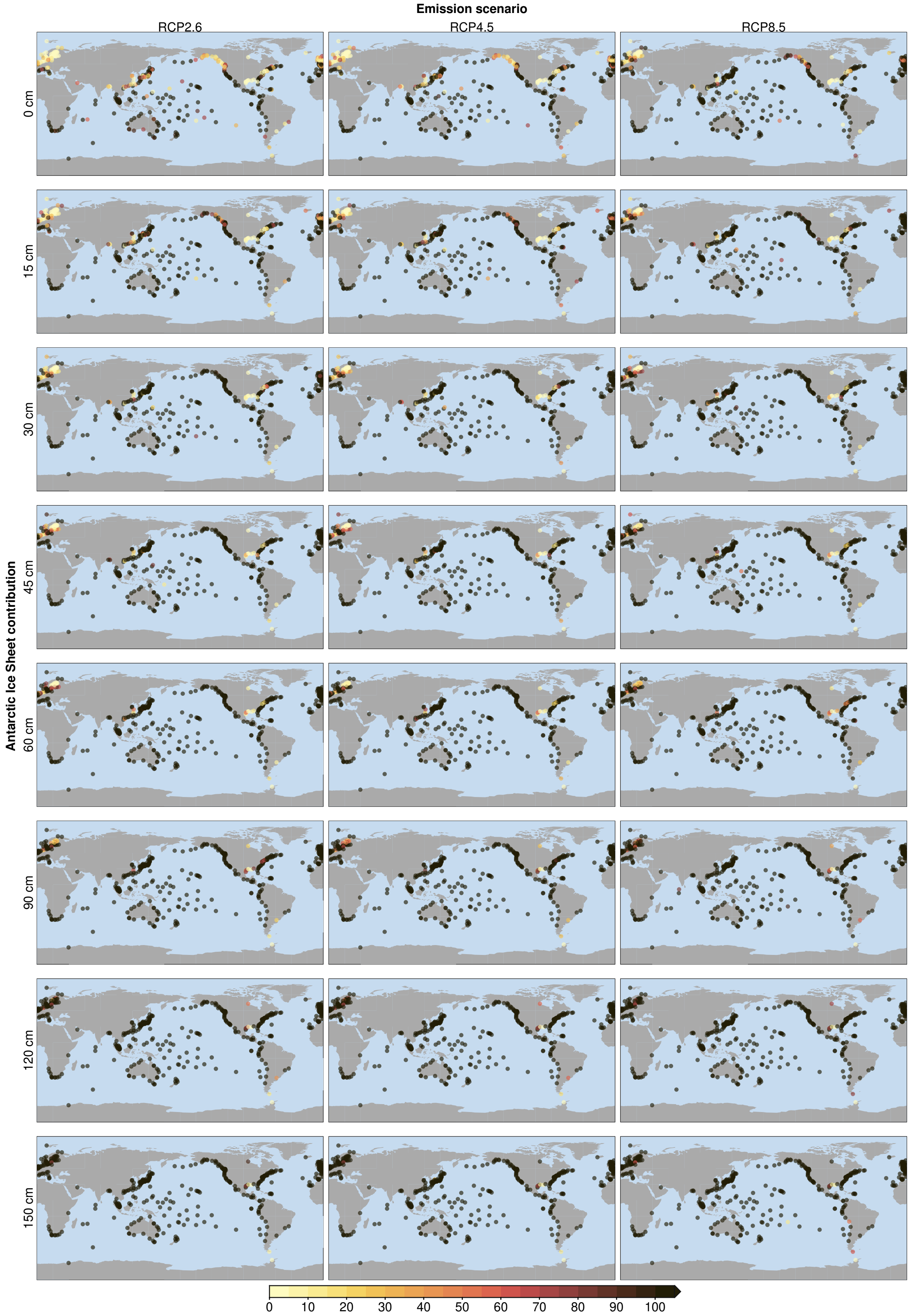

Supplementary Figure 7. Frequency amplification factors for the present-day 100-year ESL event in 2081-2100 for the RCP scenarios (left to right) and AIS contributions (top to bottom).

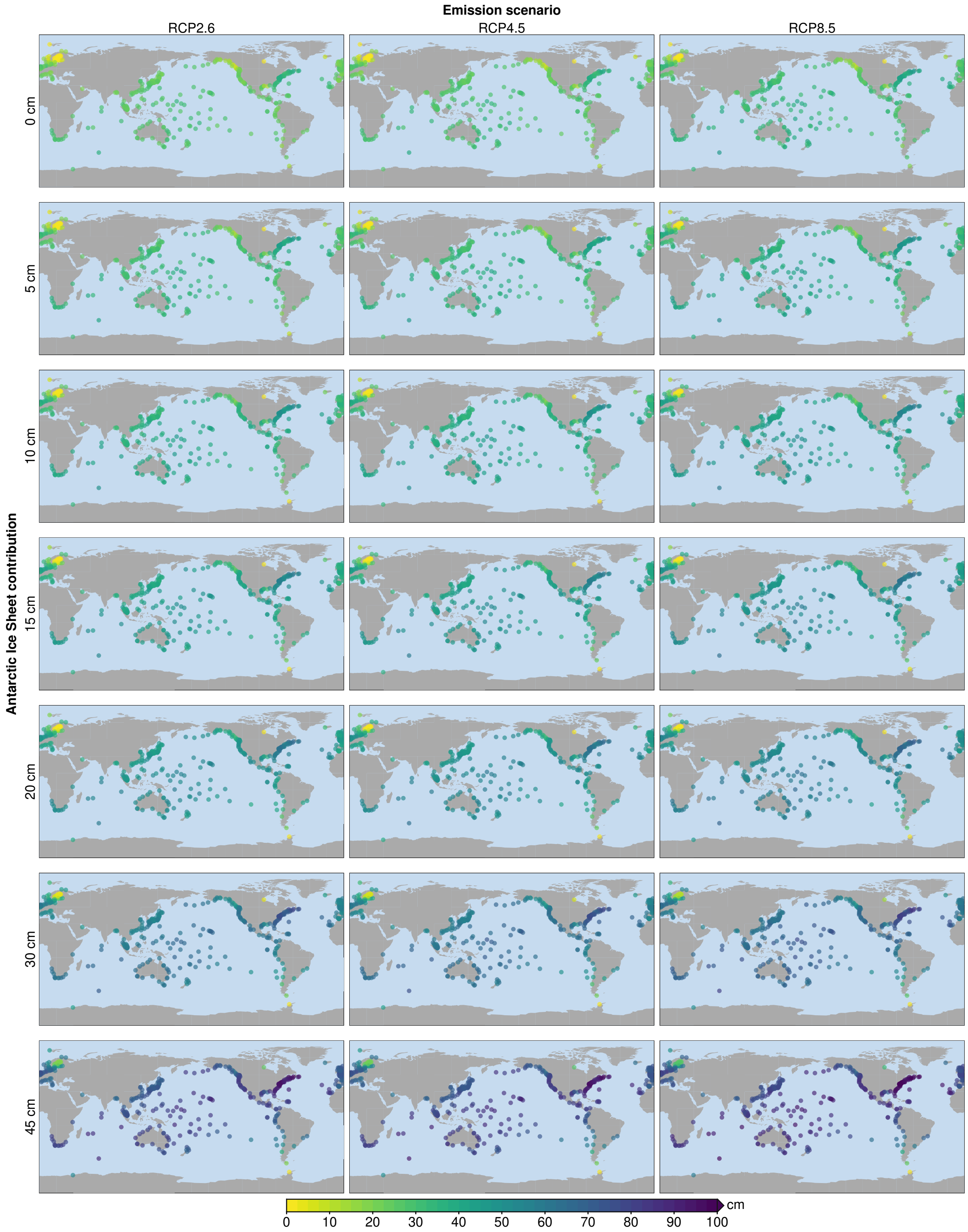

Supplementary Figure 8. Allowances for the present-day 100-year ESL event in 2046-2065 for the RCP scenarios (left to right) and AIS contributions (top to bottom).

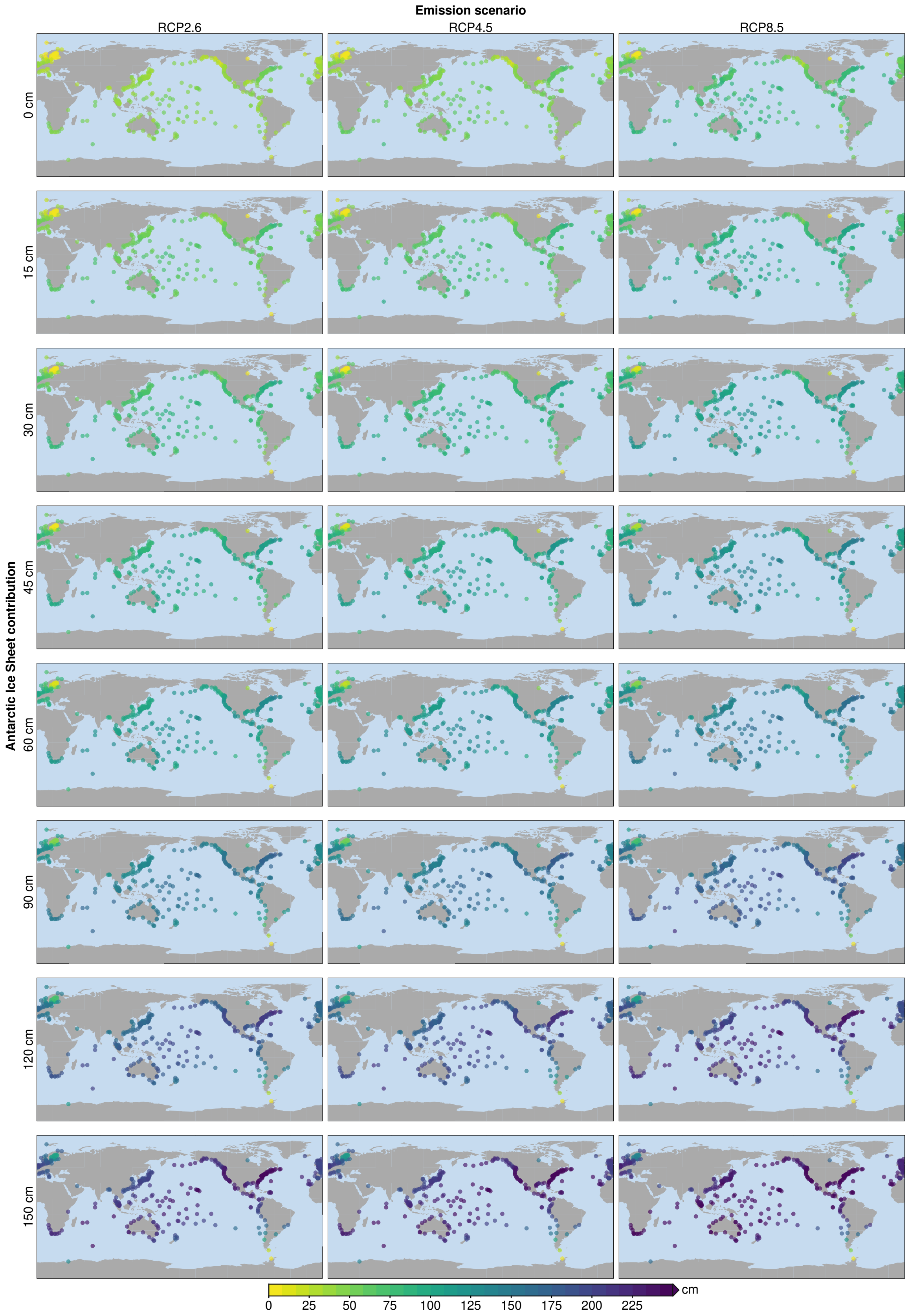

Supplementary Figure 9. Allowances for the present-day 100-year ESL event in 2081-2100 for the RCP scenarios (left to right) and AIS contributions (top to bottom).
